# Positive lymph node ratio is an index in predicting prognosis for remnant gastric cancer with insufficient retrieved lymph node in R0 resection

**DOI:** 10.1038/s41598-021-81663-0

**Published:** 2021-01-21

**Authors:** Honghu Wang, Hao Qi, Xiaofang Liu, Ziming Gao, Iko Hidasa, Ailixier Aikebaier, Kai Li

**Affiliations:** Department of Surgical Oncology, First Affiliated Hospital of China Medical University, Shenyang, 110001 Liaoning China

**Keywords:** Cancer, Oncology

## Abstract

The staging system of remnant gastric cancer (RGC) has not yet been established, with the current staging being based on the guidelines for primary gastric cancer. Often, surgeries for RGC fail to achieve the > 15 lymph nodes needed for TNM staging. Compared with the pN staging system, lymph node ratio (NR) may be more accurate for RGC staging and prognosis prediction. We retrospectively analyzed the data of 208 patients who underwent R0 gastrectomy with curative intent and who have ≤ 15 retrieved lymph nodes (RLNs) for RGC between 2000 and 2014. The patients were divided into four groups on the basis of the NR cutoffs: rN0: 0; rN1: > 0 and ≤ 1/6; rN2: > 1/6 and ≤ 1/2; and rN3: > 1/2. The 5-year overall survival (OS) rates for rN0, rN1, rN2, and rN3 were 84.3%, 64.7%, 31.5%, and 12.7%, respectively. Multivariable analyses revealed that tumor size (p = 0.005), lymphovascular invasion (p = 0.023), and NR (p < 0.001), but not pN stage (p = 0.682), were independent factors for OS. When the RLN count is ≤ 15, the NR is superior to pN as an important and independent prognostic index of RGC, thus predicting the prognosis of RGC patients more accurately.

## Introduction

Remnant gastric cancer (RGC) is an unusual but important type of gastric cancer (GC) comprising malignant tumors in the remnant stomach after subtotal distal gastrectomy, regardless of whether the initial disease is benign or malignant as well as the duration between the initial operation and diagnosis^1^. RGC is rare, accounting for only approximately 1–8% of all GC cases^2^, but its incidence has been rising owing to the increase in early GC incidence, which, in turn, is due to improvements in medical examination and the prolonged survival of patients with primary GC^3,4^. Despite its difference from other GC types, RGC staging is still based on the TNM staging system of GC. However, a retrieved lymph node (RLN) count of ≤ 15 may lead to false staging and affect treatment plans and prognosis. Furthermore, most RGC patients are in the advanced stage because of the lack of specific symptoms during the early stage, which leads to a lower radical resection rate and poor prognosis^5^. The metastatic lymph node (LN) ratio (NR) is reported to be more suitable for primary GC staging compared to a positive LN count, especially when the RLN is low^6^. This study aimed to develop a more accurate staging system for RGC. We hypothesize that NR would be more exact than the UICC/AJCC pN stage (8th edition) for RGC staging, which we validated through a retrospective analysis.

## Methods

### Patients

Written informed consent was obtained from all the patients. The study was approved by the Research Ethics Committee of the Affiliated Hospital of China Medical University and all experiments were performed in accordance with relevant guidelines and regulations. We established a retrospectively maintained database of all patients with RGC who underwent surgery at the Department of Oncology, First Affiliated Hospital of China Medical University between January 2000 and December 2014. Of them, we evaluated 243 patients who underwent radical (R0) surgery (with D2 lymphadenectomy) for RGC. However, only 7 of these patients had lymph node metastases > 15, and considering that this number of cases is too small to be representative, we finally excluded them. Surgery had been performed in all patients using an open approach, with the second surgery being total gastrectomy with Roux-en-Y anastomosis. None of the patients received neoadjuvant chemotherapy. All of the study participants were followed up through telephone or written communications until death or end of the study period (December 31, 2015). We declared that the informed consent from patients has been obtained prior to our study. As some patients invovled in our study died during the follow up, we obtained the informed consent from their guardians.

The median and mean follow-up periods were 60 and 83.15 months (range 1–346 months), respectively. In total, 11 patients were lost to follow-up, and 17 died during the postoperative period. Finally, 208 patients were included in the analysis. Postoperative chemotherapy regimens included 5-fluorouracil (5-FU) plus cisplatin, 5-FU plus mitomycin and epirubicin, 5-FU plus leucovorin and cisplatin, 5-FU plus cisplatin and epirubicin, and cisplatin plus oxaliplatin.

### Data collection

Data of the following variables were collected: (1) gender (male or female); (2) age (≤ 60 or > 60 years); (3) tumor size (≤ 4 cm or > 4 cm); (4) macroscopic serosal classification (normal, reactive, nodular, tendonoid, and color-diffused type); (5) Borrmann type (Borrmann 1, 2, 3, and 4); (6) depth of tumor invasion according to the 8th UICC/AJCC (T1, T2, T3, T4a, or T4b); (7) Lauren’s classification (intestinal or diffuse); (8) histology (differentiated or undifferentiated); (9) lymphovascular invasion; (10) number of positive LNs according to the 8th UICC/AJCC (N0, N1, N2, or N3); (11) NR (0, 0–1/6, 1/6–1/2, > 1/2); and (12) postoperative adjuvant therapy.

### Retrieved lymph node categories

The best cut-off approach was used to determine LN ratio categories, with patient survival (log-rank statistic) as the dependent variable. Accordingly, 1/6 and 1/2 were identified as the best cutoff for discriminating differential prognoses in RGC patients. Thus, patients were divided into four groups: rN0, NR = 0; rN1, > 0 and ≤ 1/6; rN2, > 1/6 and ≤ 1/2; and rN3, > 1/2. Cases in which the retrieved LN was 0 were considered as N0 and NR = 0.

### Statistical analyses

All statistical analyses were performed using SPSS software version 20.0. We used the Kaplan–Meier method to determine overall survival (OS) and the log-rank test for comparison. Cox’s proportional hazard model (forward-stepwise method) was used for the multivariate analysis to determine the independent factors for prognosis prediction. We divided the multivariate analysis into two steps. In step 1, we analyzed all significantly important prognostic factors using multivariate analysis, with the exception of the revised NR stage. In step 2, we performed multivariate analysis with consideration of all significantly important prognostic factors, including the revised NR stage. Two-tailed p-values and differences were considered significant at p < 0.05.

## Results

### Clinicopathological characteristics and prognostic factors

The clinicopathological characteristics of the 208 patients are summarized in Table 1. The NR distribution was as follows: rN0, 108 patients (36.6%); rN1, 51 patients (17.3%); rN2, 73 patients (24.7%); rN3, 63 patients (21.4%). Univariate analysis showed that tumor size (≤ 4 cm vs. > 4 cm, p < 0.001), Borrmann type (high vs. low, p < 0.001), histology (differentiated vs. undifferentiated, p = 0.011), Lauren classification (intestinal vs. diffused, p = 0.001), lymphovascular invasion (positive vs. negative, p < 0.001), pT stage (early vs. advanced, p < 0.001), pN stage (early vs. advanced, p < 0.001), NR (high vs. low, p < 0.001), and postoperative adjuvant therapy (yes vs. no, p = 0.031), but not gender, age, and macroscopic serosal classification, (p = 0.113, 0.393, and 0.084, respectively), were significantly correlated with OS after curative surgery (Table 2). The mean survival of each pN stage was 131.34 ± 6.49, 73.14 ± 7.25, 36.37 ± 3.72, and 25.7 ± 5.78 months, whereas the mean survival for each NR stage was 131.34 ± 6.49, 91.35 ± 9.48, 46.96 ± 5.65, and 33.33 ± 2.36 months, respectively.

**Table 1:** Descriptive data of the patients involved in this cohort (n = 208).

Patient characteristics	n	%
**Age (years)**		
≤ 60	109	52.4
> 60	99	47.6
**Gender**		
Male	166	79.8
Female	42	20.2
**Tumor size (cm)**		
≤ 4 cm	83	39.9
> 4 cm	125	60.1
**Borrmann type**		
Borrmann 1	9	4.3
Borrmann 2	53	25.5
Borrmann 3	128	61.5
Borrmann 4	18	8.7
**Macroscopic serosal classification**		
Normal	12	5.8
Reactive	31	14.9
Nodular	71	34.1
Tendonoid	70	33.7
Color-diffused	24	11.5
**Histology**		
Undifferentiated	116	55.8
Differentiated	92	44.2
**Lauren’s classification**		
Intestinal	112	53.8
Diffuse	96	46.2
**Lymphovascular invasion**		
Positive	43	20.7
Negative	165	79.3
**8th UICC/AJCC pT stage**		
T1	8	3.9
T2	35	16.8
T3	103	49.5
T4a	33	15.9
T4b	29	13.9
**8th UICC/AJCC pN stage**		
N0	76	36.5
N1	66	31.7
N2	59	28.4
N3a/3b	7	3.4
**NR**		
0	76	36.5
> 0, ≤ 1/6	36	17.3
> 1/6, ≤ 1/2	52	25
> 1/2	44	21.2
**Postoperative adjuvant therapy**		
Yes	33	15.9
No	175	84.1

*UICC* union for international cancer control, *AJCC* American joint committee on cancer, *NR* lymph node ratio.

**Table 2:** Univariate analysis and two-step multivariate analysis for overall survival.

	Univariate analysis				Multivariate analysis step 1^a^			Multivariate analysis step 2^b^		
	n	5-YSR%	× 2	p	HR	95% CI	p	HR	95% CI	p
**Age (years)**			0.731	0.393						
≤ 60	109	54.8								
> 60	99	50.0								
**Gender**			2.510	0.113						
Male	166	50.2								
Female	42	61.7								
**Tumor size (cm)**			19.486	0.000			0.007			0.005
≤ 4 cm	83	66.9								
> 4 cm	125	42.9			1.687	1.151–2.471		1.708	1.171–2.493	
**Borrmann type**			19.795	0.000						
Borrmann 1	9	84.6								
Borrmann 2	53	67.6								
Borrmann 3	128	47.8								
Borrmann 4	18	26.9								
**Macroscopic serosal classification**			8.226	0.084						
Normal	12	64.7								
Reactive	31	59.1								
Nodular	71	56.4								
Tendonoid	70	48.5								
Color-diffused	24	38.2								
**Histology**			6.422	0.011						
Undifferentiated	116	46.1								
Differentiated	92	60.8								
**Lauren’s classification**			10.096	0.001						
Intestinal	112	61.6								
Diffuse	96	41.9								
**Lymphovascular invasion**			12.524	0.000			0.002			0.023
Positive	43	36.1								
Negative	165	56.8			0.542	0.370–0.792		0.645	0.442–0.941	
**8th UICC/AJCC pT stage**			40.325	0.000						
T1	8	100.0								
T2	35	69.4								
T3	103	55.5								
T4a	33	36.2								
T4b	29	26.8								
**8th UICC/AJCC pN stage**			103.539	0.000			0.000			
N0	76	84.3								
N1	66	48.4			3.668	2.094–6.425				
N2	59	21.4			8.186	4.748–14.114				
N3a/3b	7	10.0			9.960	4.367–22.716				
**NR**			125.406	0.000						0.000
0	76	84.3								
> 0, ≤ 1/6	36	64.7						2.396	1.234–4.654	
> 1/6, ≤ 1/2	52	31.5						6.102	3.484–10.689	
> 1/2	44	12.7						9.867	5.641–17.259	
**Postoperative adjuvant therapy**			4.629	0.031						
Yes	33	66.0								
No	175	50.0								

*5-YSR* 5-year survival rate, *UICC* union for international cancer control, *AJCC* American joint committee on cancer, *HR* hazard ratio, *CI* confidence interval, *NR* lymph node ratio.

^a^Step 1, with consideration of all significantly important prognostic factors in the univariate analysis, with the exception of the revised NR stage.

^b^Step 2, with consideration of all significantly important prognostic factors in the univariate analysis, including the revised NR stage.

### Multivariate analyses

To determine which is more accurate between pN stage and NR stage as a prognostic index for the OS of RGC patients, we conducted a two-step multivariate analysis of prognosis. In step 1, we performed a multivariate analysis of all the important prognostic factors (tumor size, Borrmann type, histology, Lauren classification, lymphovascular invasion, pT stage, pN stage, postoperative adjuvant therapy), except for NR staging. We found that tumor size (p = 0.007), lymphovascular invasion (p = 0.002), and pN stage (p < 0.001) were independent factors predicting prognosis (Table 2). However, NR stage was superior to pN stage (UICC/AJCC) as an independent prognostic indicator (Table 2), as determined in step 2 of the analysis. Moreover, pT stage was remarkably not an independent prognostic index for RGC patients, according to our analysis.

### Migration

We further calculated the migration among the different stages according to the new NR stages. We found that 25 and 5 cases of pN1 were re-divided into rN2 and rN3, and 33 cases of pN2 were re-divided into rN3, respectively (Table 3A). Migration of the corresponding modified TNM stage using NR (mTNM-NR) staging was also observed, as shown in Table 3B.

**Table 3:** Migration between numeric-based versus lymph node ratio-based staging systems for remnant gastric cancer.

(A)	pN (8th UICC/AJCC)	NR
pN1 vs rN1	66	36
pN2 vs rN2	59	51
pN3 vs rN3	7	45

(B)	TNM	mTNM-NR
IA	1	1
IB	23	23
IIA	50	46
IIB	45	29
IIIA	46	38
IIIB	25	46
IIIC	18	25

(A) pN (8th UICC/AJCC) vs. NR staging systems, (B) TNM (8th UICC/ AJCC) vs. mTNM-NR staging systems.

*UICC* union for international cancer control, *AJCC* American joint committee on cancer, *NR* lymph node ratio.

**Figure 1:**
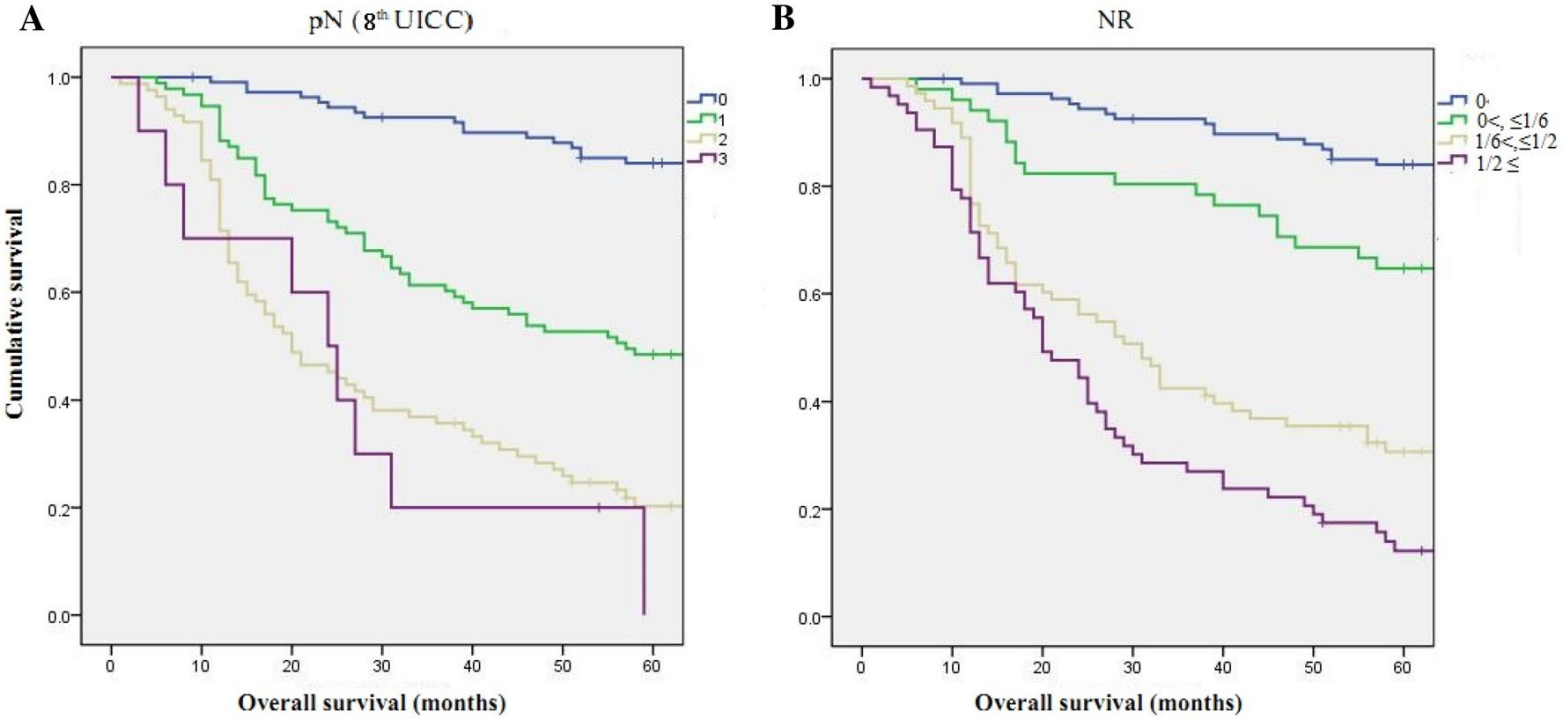
Comparison of survival curves according to the pN and NR stages (both p < 0.001). (**a**) Survival curves of patients with various pN stages. (**b**) Survival curves of patients with various NR stages. *NR* lymph node metastatic ratio.

**Figure 2:**
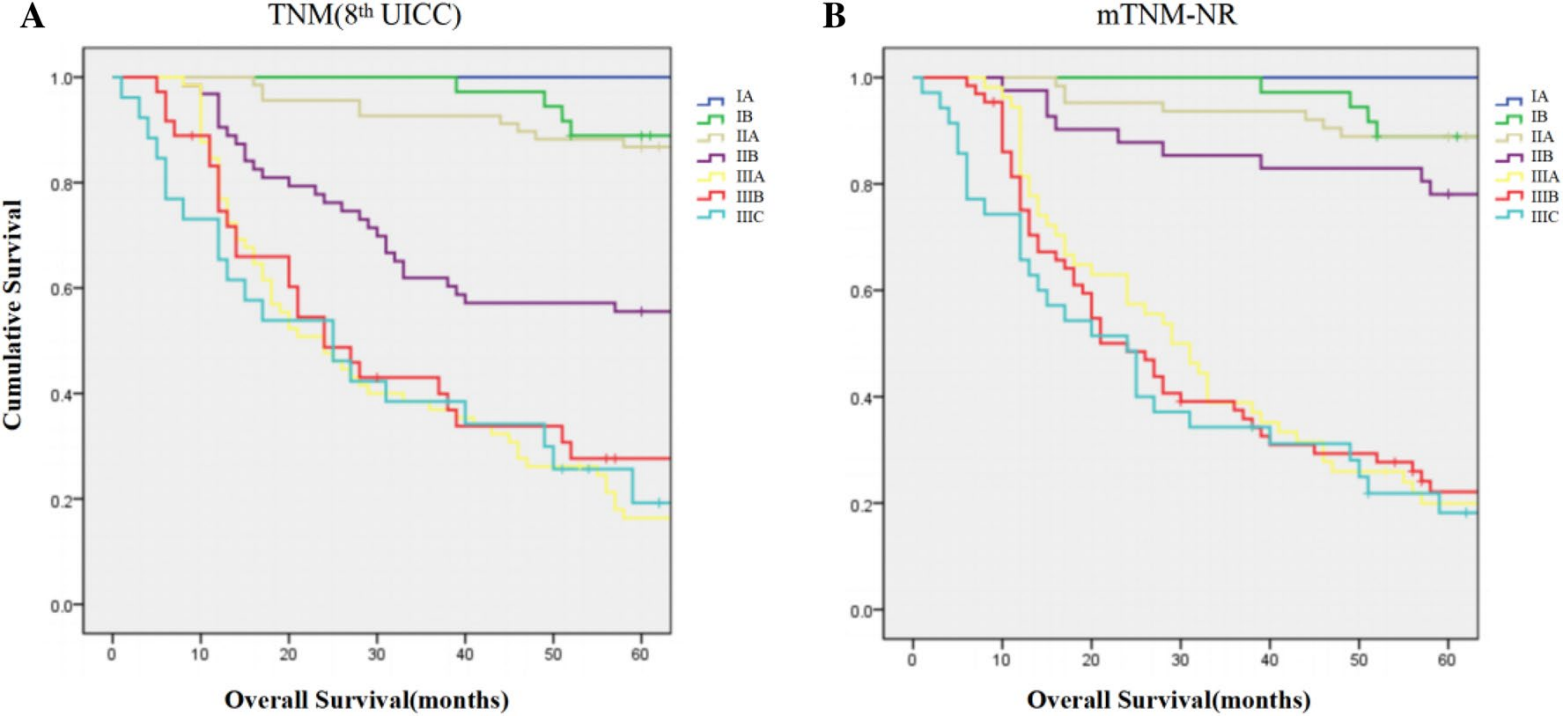
Kaplan–Meier curves. (**a**) Kaplan–Meier curves according to the TNM (8th UICC/AJCC) classification system. (**b**) Kaplan–Meier curves according to the mTNM-NR classification system. *UICC* union of international cancer control, *AJCC* American joint committee on cancer, *NR* lymph node metastatic ratio, *mTNM-NR* modified TNM stage using NR.

As shown in Fig. 1, there was a significant difference in prognosis between pN and NR stages among the RGC patients. According to the analysis, there was no significant difference between pN2 and pN3 (p = 0.511), but there was a significant difference between rN2 and rN3 (p = 0.034) in their predictive capability for the prognosis of RGC patients. In addition, we presented the Kaplan–Meier curves based on the TNM staging (UICC/AJCC 8th edition) and mTNM-NR staging system as shown in Fig. 2.

### Correlations analysis

We further analyzed the correlation between NR and other clinicopathologic features. As shown in Table 4, there was significant positive correlation between NR and the following parameters: tumor size (p = 0.000), Borrmann type (p = 0.000), macroscopic serosal classification (p = 0.002), lauren’s classification (0.009), and pT stage (0.000). And a negative correlation was shown between NR and histology (p = 0.007), lymphovascular invasion (0.003).

**Table 4:** Correlation between NR and other clinicopathologic parameters.

	NR	
	Pearson correlation	P (two-tailed)
Age	0.084	0.151
Gender	0.018	0.759
Tumor size	0.222	0.000
Borrmann type	0.249	0.000
Macroscopic serosal classification	0.179	0.002
Histology	− 0.158	0.007
Lauren’s classification	0.151	0.009
Lymphovascular invasion	− 0.173	0.003
8th UICC/AJCC pT stage	0.322	0.000
Postoperative adjuvant therapy	− 0.083	0.155

*Correlation is significant at the 0.01 level (2-tailed).

### Characteristic of lymph nodes metastasis

The total number of lymph nodes retrieved was 1152, of which 426 were metastases. We present the distribution and basic statistics of the total retrieved LN numbers with metastatic LN numbers in Table 5.

**Table 5:** Characteristic of lymph nodes metastasis.

Regional lymph node	Retrieved LNs	Metastatic LNs	Rate of LN metastasis (%)
No. 1 right cardiac	176	64	36.4
No. 2 left cardiac	132	37	28.0
No. 3 lesser curvature	204	107	52.4
No. 4 greater curvature	114	48	42.1
No. 7 left gastric artery	94	30	31.9
No. 8 common hepatic artery	58	9	15.5
No. 9 celiac artery	38	14	36.8
No. 10 splenic hilum	98	42	42.9
No. 11 splenic artery	76	26	34.2
No. 12 ligamentum hepatoduodenale	44	7	15.9
No. 14 mesenteric roots	24	7	29.2
No. 20 infra-diaphragm around the esophagus hilus	94	35	37.2

## Discussion

While RGC has been extensively studied, its staging system is yet to be established. Therefore, RGC staging generally follows the TNM staging of primary GC^1,7^. In the 8th UICC/AJCC TNM staging system, the amount of metastatic LNs is used to determine the LN stage, and the number of RLNs must be > 15 to arrive at a more accurate LN staging. However, this appears to be inappropriate for RGC where RLNs can be < 16, particularly in the case of radical lymphadenectomy for initial malignant disease^8^. Previous studies have shown that LN metastasis is an independent risk factor in RGC local recurrence. Moreover, complete resection and LN dissection are key to successful radical gastrectomy for RGC. Thus, appropriate LN staging can accurately indicate patient prognosis, guiding further treatment. By contrast, inadequate LN dissection can lead to residual cancer cells, which results in a higher recurrence rate after surgery. Ultimately, it remains unclear whether the 8th UICC/AJCC pN staging can accurately assess the prognosis of RGC patients.

NR is the ratio of the amount of positive LN to that of the RLN. Despite the same number of metastases, cases with higher RLN and thus lower NR have better prognosis^9^. Due to the common occurrence of low RLN cases during RGC resection, we hypothesized that NR was more accurate than the present pN stage, which was confirmed by our results. The NR classification system stratifies each stage better; in our analysis, we used a multiple Cox regression model to select the NR stage as a substitute for the pN stage. In addition, lymphovascular invasion and tumor size were also proven to be independent risk indexes for prognosis.

The NR system has obvious advantages and attractive prospects for clinical application in RGC. Dapeng et al. showed that NR plays a role as an independent prognostic factor after radical operation for GC^10^. Lee et al. also suggested that among patients with stage IIIA GC, the prognosis differed between those with RLN ≤ 15 and > 15^11^. Ichikura’s study found that the amount of positive LNs is closely related to the RLN^12^. Both a low and exceedingly high RLN may lead to inaccurate staging, leading to inaccurate assessments of patient prognosis. Therefore, some researchers suggested using the LN metastasis rate for LN staging as it can reflect both the amount of dissected LN and positive LN^13^. On the other hand, there is no consensus on the prognostic value of NR in RGC patients.

Lu et al. reported that the tumor size may be more hierarchical than the pN (7th UICC) stage system in RGC^14^. Similarly, we found that tumor size is an independent risk factor of prognosis. RGC is complicated to manage because of its diversity, and the initial operation significantly influences the second operation. Most researchers stipulate that initial gastrectomy alters the lymph flow from the remnant stomach and LN metastasis due to reconstruction and adhesion, leading to the occurrence of RGC^15,16^. Several authors have reported that the risk of RGC was higher after Billroth II reconstruction. Gandolfi et al. found that Billroth II was the most commonly used approach (95%) for reconstruction during the initial surgery of RGC patients^17^. With respect to the type of surgical anastomosis, it was reported that compared with Billroth I, Billroth II surgery increased the risk of RGC by four times^18^. Xiang et al. found that RGC mostly occurred at the anastomotic sites, and that LN metastasis of the jejunal mesentery and mesenteric root was universal, particularly in patients who underwent Billroth II anastomoses^16^. In addition, our results demonstrate that T stage was not an independent prognostic factor for patients with RGC. We speculated that RCG is characterized by alterations in the normal anatomic lymphatic flow of the stomach and its association with the surrounding tissue. Adhesion after primary operation may influence the progression of cancer and the infiltration of surrounding organs, especially for RGC at the anastomosis site. In our study, we enrolled 208 RGC cases, there were mainly cases with the depth of invasion at T2 (n = 35, 16.8%), T3 (n = 103, 49.5%) and T4 (n = 62, 29.8%). Only 8 cases were T1 stage (3.9%). Alberto et al.^19^ reported that RGC with pT1 stage had lower rate of lymph node metastasis. Once the depth of invasion was T2 or above, the rate of LN metastasis increased significantly. The lymph node dissection for RGC should be extended beyond the initial operation. A high incidence of LN metastasis in the No. 10–14, 20, 110, 111 was found in the patients who had previously undergone gastric reconstruction. All the regional lymph nodes around the stomach that were not cleared in initial operation should be dissected. The LN metastasis rate was 42.9%, 34.2%, 15.9%, 29.2% and 37.2% in No. 10, 11, 12, 14 and 20 in our study. Therefore, it is not at all surprised that N categories replaced T categories being an independent risk factor for prognosis of RGC. In addition, the small sample size may contribute this result. We also observed LN metastasis of the jejunal mesentery during RGC surgeries (n = 2). However, this needs to be further validated and analyzed in future research.

In contrast to our findings, some studies reported that only pT and pN are the prognostic factors of RGC. Nakagawa et al. performed a similar study but obtained a contrasting conclusion, stating that NR was not better to pN (7th UICC)^4^. The contrasting findings may be attributed to the different NR cut-off points and the eligibility criteria between the current study and that by Nakagawa et al. In Nakagawa’s study, the NR cut-offs were 0; > 0, ≤ 0.1; > 0.1, ≤ 0.4; and < 0.4. When Marchet analyzed the clinical data of 1853 patients with gastric cancer, NR was divided into 0%, 1–9%, 10–25%, and > 25%^20^. Satio et al. divided NR stages into 0%, > 5%, 5–10%, 10–20%, 20–30%, and > 30%, and found that NR was an independent prognostic factor in patients with gastric cancer; however, the number of positive LNs was not an independent factor^21^. Bando et al.^22^ studied 650 GC patients who had > 15 LN dissections after operation. Their NR staging was divided into 0%, < 10%, 10–25%, and > 25%, and they found that NR staging was more effective than the JGCA LN staging. Moreover, Sun et al. identified the following best-fit cut-off values: rN0: 0%; rN1: 1–20%; rN2: 21–50% and rN3: > 50%, proving the advantage of NR stage in minimizing stage migration for GC patients with insufficient number or level of retrieved LNs^23^. Furthermore, few scholars have elaborated on the method of dividing NR staging nodes. There is no international consensus; hence, further research is needed to determine the appropriate staging nodes. In our study, we identified the NR stages as rN0: 0; rN1: > 0 and ≤ 1/6; rN2: > 1/6 and ≤ 1/2; and rN3: > 1/2.

Moreover, a significant portion of patients included in our study underwent Billroth I and Roux-Y during the initial surgical reconstruction (n = 41, 21%). However, we only focused on those who underwent Billroth II. In addition, we included cases with RLN ≤ 15, and excluded the very few cases with RLN > 16. By contrast, Nakagawa et al. included both cases of RLN ≤ 15 and > 15. One study showed that if the number of RLN after radical gastrectomy was > 15, then the prognostic value of NR for patients with GC was worse than that of N stage^24^, supporting our findings that NR stage can predict the prognosis of RGC patients more accurately than pN stage.

Our study has some limitations. First, it spanned a relatively long period of 15 years. Continuous improvements in surgical instrumentation and chemotherapy regimens may have affected the outcomes. We also evaluated a specific sample population, and thus, our findings need to be further validated in other types of RGC patients and cohorts, particularly in the Western population. Because the number of cases with RLN > 15 is too small to be representative, our study included only samples with ≤ 15 RLN. This may limit the possibility of the NR staging system being developed as a standard staging system in clinical practice, and our findings should hence be verified by further big data studies. We suggest that for RGC with RLN > 15, the original pN stage (8th edition) can be used.

In conclusion, the metastatic LN ratio is an independent prognostic index for patients with RGC. When the amount of RLN is ≤ 15, the NR stage can more accurately predict the prognosis of patients with RGC than the pN stage, facilitating the selection of better treatment options.

## Data Availability

At present, the datasets generated during and analyzed during the current study are being used in another research; hence, these are not publicly available but could be available from the corresponding author on reasonable request.
